# Predictive Insights Into Bioactive Compounds from Streptomyces as Inhibitors of SARS-CoV-2 Mutant Strains by Receptor Binding Domain: Molecular Docking and Dynamics Simulation Approaches

**DOI:** 10.5812/ijpr-150879

**Published:** 2024-12-08

**Authors:** Hourieh Kalhor, Mohammad Hossein Mokhtarian, Hamzeh Rahimi, Behzad Shahbazi, Reyhaneh Kalhor, Tahereh Komeili Movahed, Hoda Abolhasani

**Affiliations:** 1 Cellular and Molecular Research Center, Qom University of Medical Sciences, Qom, Iran; 2 Department of Microbiology and Immunology, Faculty of Veterinary Medicine, Garmsar Branch, Islamic Azad University, Garmsar, Iran; 3 Department of Neurosurgery, City of Hope, California, USA; 4 School of Pharmacy, Semnan University of Medical Sciences, Semnan, Iran; 5 Nervous System Stem Cells Research Center, Semnan University of Medical Sciences, Semnan, Iran; 6 Department of Pharmacology, School of Medicine, Qom University of Medical Sciences, Qom, Iran

**Keywords:** SARS-CoV-2, ACE2, Stambomycin B, Molecular Docking, Molecular Dynamics Simulation

## Abstract

**Background:**

The receptor-binding domain (RBD) of the spike protein of SARS-CoV-2 interacts with the angiotensin-converting enzyme 2 (ACE2) receptor in humans. To date, numerous SARS-CoV-2 variants, particularly those involving mutations in the RBD, have been identified. These variants exhibit differences in transmission, pathogenicity, diagnostics, and vaccine efficacy.

**Objectives:**

Although therapeutic agents are currently available to inhibit SARS-CoV-2, most provide supportive and symptomatic relief. Moreover, different variants may exhibit resistance to these treatments. This study aimed to identify a potential compound with favorable antiviral effects against SARS-CoV-2 variants.

**Methods:**

The study explored drug discovery through structure-based virtual screening of natural products (NPs) from the StreptomeDB database, targeting the ACE2-binding pocket of the SARS-CoV-2 RBD protein. The analysis included the wild-type protein (PDB ID: 6VW1) as well as the Alpha, Beta, Delta, Lambda, Omicron/BA.1, and Omicron/BA.2 variants.

**Results:**

In silico screening identified ‘Stambomycin B’ as a potential compound with the highest binding affinity. Molecular dynamics simulations of the complexes, conducted over 100 ns, confirmed the prediction that ‘Stambomycin B’ could inhibit different SARS-CoV-2 variants effectively.

**Conclusions:**

This study concludes that ‘Stambomycin B’, a macrolide compound produced by *Streptomyces ambofaciens*, may be a candidate NP for effectively combating all mutants that occur in the binding of SARS-CoV-2 RBD to ACE2, even those that may arise in the future.

## 1. Background

SARS-CoV-2 is a Betacoronavirus that can cause moderate to severe respiratory diseases in humans (1-3). The SARS-CoV-2 genome comprises both structural and non-structural proteins (4). Among the structural proteins is the spike protein (S protein), which facilitates human coronavirus infection primarily through its interaction with human angiotensin-converting enzyme 2 (ACE2) (5, 6).

The S protein consists of two subunits, S1 and S2. The receptor-binding domain (RBD), which binds to the ACE2 receptor, is located in the S1 subunit within the N-terminal domain, while the S2 subunit mediates fusion of the virus with the human cell membrane (7, 8). Continuous mutations in the virus can lead to the emergence of new variants that differ in characteristics such as transmission, pathogenicity, diagnostics, and vaccine efficacy (9, 10).

Each new variant is defined by specific mutations in its spike proteins, particularly in the RBD. Notably, these mutations significantly influence the binding affinity between the RBD and ACE2, as well as the virus's ability to evade the immune response (11-15). However, this interaction mechanism between the RBD and ACE2 provides a valuable basis for developing compounds that can inhibit viral entry into host cells, thereby reducing the likelihood of future infections (16, 17).

The major variants identified to date include Alpha (B.1.1.7), Beta (B.1.351), Gamma (P.1), Delta (B.1.617.2), and Omicron (B.1.1.529) (11).

Several studies have employed computational screening techniques, including molecular docking and structural dynamics, to explore the potential of various compounds targeting different SARS-CoV-2 proteins. These proteins include non-structural proteins 1, 15, and 16, RNA-dependent RNA polymerase, and the spike (S) protein (18-22).

For instance, a recent study reported that the compounds 9‴-Methyllithospermate, Epimedin A, Pentagalloylglucose, and Theaflavin-3-gallate exhibit strong binding affinity with the SARS-CoV-2 RBD (22). Similarly, other computational research demonstrated that two FDA-approved drugs, Atovaquone (ATV) and Praziquantel (PRZ), have the potential to inhibit the wild-type, Delta, Delta Plus, and Lambda variants of the S protein RBD (23).

In our previous study, we conducted structure-based virtual screening of FDA databases to identify lead drugs targeting the ACE2 binding pocket of the SARS-CoV-2 S protein. From this analysis, we identified Diammonium Glycyrrhizinate, Digitoxin, Ivermectin, Rapamycin, Rifaximin, and Amphotericin B as compounds with highly favorable characteristics, highlighting their potential as COVID-19 therapies (24).

Furthermore, secondary metabolites found in rose water—such as eugenol, alpha-terpineol, geraniol, citronellol, phenylethyl alcohol, nerol, and linalool—have been noted as potential inhibitors of the SARS-CoV-2 S protein (25).

Although therapeutics against SARS-CoV-2 are currently available, many variants have shown resistance to these treatments. Consequently, it is essential to develop new drugs and investigate potential pharmacological targets and lead compounds that can swiftly counteract and prevent SARS-CoV-2 variants. The exploration of microbial metabolites as potential therapeutic candidates for virus-mediated diseases has garnered significant attention in scientific research (17).

The use of microbial metabolites for antiviral purposes is gaining increasing popularity in biotechnology (26-29). Numerous DNA and RNA viruses have been reported to be susceptible to the antiviral effects of various microbial metabolites (30-33). Among microbial sources, *Streptomyces* spp. are recognized as highly resilient and effective organisms (34). This genus of Gram-positive actinobacteria is predominantly found as saprophytes in soil (35).

Streptomyces are renowned for their abundant production of secondary metabolites, many of which exhibit significant biological activity. They are considered potential inhibitors due to their capacity to generate diverse chemical precursors and various molecular scaffolds. Notably, approximately 80% of all known microbial bioactive compounds are derived from the Streptomyces genus, which exhibits antibacterial, antimetabolite, anticancer, antifungal, and anti-inflammatory activities. This genus produces antibiotics such as tetracycline, daptomycin, and chloramphenicol (36), antiparasitic agents, like ivermectin, immunosuppressants including rapamycin, and lipase inhibitors such as lipstatin (35, 37, 38).

In addition to these applications, Streptomyces also produce bioactive compounds with antiviral properties. For instance, aristeromycin, a compound with broad-spectrum antiviral activity, has been isolated from *Streptomyces citricolor* (39, 40). Furthermore, Omicsynin B4, a pseudo-tetrapeptide derived from the *Streptomyces* sp. 1647 strain, has demonstrated its ability to inhibit coronaviruses, including HCoV-229E, HCoV-OC43, and even SARS-CoV-2, across various cell lines. This compound has been reported to effectively inhibit the entry of coronaviruses into host cells (41).

Consequently, *Streptomyces* may represent a promising resource for the development and production of novel natural compounds capable of inhibiting viral infections such as SARS-CoV-2 (42).

## 2. Objectives

This study aimed to develop and identify a potential inhibitor targeting the interaction between the SARS-CoV-2 RBD and ACE2 across key variants (wild-type, Alpha, Beta, Delta, Lambda, Omicron/BA.1, and Omicron/BA.2) using natural products (NPs) derived from *Streptomyces* spp. To achieve this, a total of 6,524 compounds from the StreptomeDB database were screened against the SARS-CoV-2 RBD using molecular docking. Ultimately, a potential lead compound was identified and evaluated for its efficacy against the wild-type strain and selected variants.

## 3. Methods

### 3.1. Selection and Preparation of the Compounds

For this study, the StreptomeDB 3.0 database (http://www.pharmbioinf.uni-freiburg.de/streptomedb), recognized as the largest library of NPs derived from *Streptomyces* spp., was utilized. This database comprises 6,524 unique compounds. The compounds were prepared using AutoDock version 4.2. The preparation process involved merging non-polar hydrogen atoms, applying Gasteiger-Marsili charges, aligning atoms to AutoDock atom types, and defining rotatable bonds. The prepared compounds were saved in PDBQT format [Protein Data Bank (PDB), Partial Charge (Q), and Atom Type (T)].

For molecular docking using Smina software (43), the PDBQT format was converted to Structure Data Format (SDF) using the open-source chemistry toolbox Open Babel (44).

### 3.2. Selection and Preparation of the Protein

Several crystallographic structures of the SARS-CoV-2 spike (S) protein have been determined, including the main protease (PDB IDs: 6W63, 6WNP, 6M03), the spike glycoprotein in the closed state (PDB ID: 6VXX), the chimeric RBD complexed with the human ACE2 receptor (PDB ID: 6VW1), the RNA-dependent RNA polymerase (PDB ID: 6M71), and the 3CL protease (3CLpro) (PDB ID: 6M2N).

This study aimed to identify potential natural compounds capable of inhibiting the interaction between ACE2 and the SARS-CoV-2 RBD protein. For this purpose, we selected the X-ray structure of the RBD complexed with the human ACE2 receptor (PDB ID: 6VW1), which was resolved at 2.68 Å (45). Based on the literature, we focused on variants with enhanced infectivity, including Alpha, Beta, Delta, Lambda, Omicron/BA.1, and Omicron/BA.2. The 3D structures of these variants were predicted from the wild-type structure (PDB ID: 6VW1) using Modeller v9.15 software (46) (Table 1). The best-scoring models were subsequently refined using the GalaxyRefine web server (47).

**Table 1.** Generated SARS-CoV-2 Receptor-Binding Domain Variants

Lineage	B.1.1.7	B.1.351	PI	B.1.617	C.37	BA.1	BA.2
**Synonyms**	UK/Alpha	South Africa/ Beta	Brazil/Gama	India/Delta	lambda	Omicron/BA.1	Omicron/BA.2
**Mutations on RBD**	Glu 484 Lys, Asn 501 Tyr, Ser 494 Pro	Glu 484 Lys, Lys 417Asn, Asn 501 Tyr	Glu 484 Lys, Asn 501Tyr, Lys 417Asn	Glu 484 Gln, Leu 452 Arg, Thr 478 Lys, Lys 417 Asn	Leu 452 Gln, Phe 490 Ser	Asn 440 Lys, Gly 446 Ser, Leu 452 Arg, Ser 447 Asn, Thr 478 Lys, Glu 484 Ala, Gln 493 Arg, Gly 496 Ser, Gln 498 Arg, Asn 501 Tyr, Tyr 505 His	Asn 440 Lys, Ser 447 Asn, Thr 478 Lys, Glu 484 Ala, Gln 493 Arg, Gln 498 Arg, Asn 501 Tyr, Tyr 505 His

Finally, the structures of the wild-type and selected variants of the SARS-CoV-2 RBD were prepared using AutoDockTools (48). During this process, water, solvent molecules, and other ligands were removed. AutoDock atom types, polar hydrogens, and partial charges were assigned to the 3D structures to prepare them for further analysis.

### 3.3. Selection and Preparation of the Binding Site

In our previous study, we analyzed the crystallographic structure of the SARS-CoV-2/ACE2 complex (PDB ID: 6VW1) using the PDBsum web tool and LigPlot+ software (49, 50). We identified a total of 16 residues—Tyr449, Tyr453, Leu455, Phe456, Ala475, Gly476, Phe486, Asn487, Tyr489, Gln493, Gly496, Gln498, Thr500, Asn501, Gly502, and Tyr505—that are involved in the binding of the SARS-CoV-2 RBD to ACE2. These residues were selected as the primary binding site residues for virtual screening.

According to previous studies, these residues were confirmed as key residues of the binding site. Based on our analysis, a grid box with dimensions of X = 26 Å, Y = 42 Å, and Z = 26 Å, and a grid spacing of 1 Å, was defined for docking studies (24).

### 3.4. Structure-Based Virtual Screening

Natural products from the StreptomeDB database and the ACE2 binding site of the SARS-CoV-2 RBD for both the wild-type and variant strains were subjected to Structure-based virtual Screening (SBVS) studies using Smina software (43). Subsequently, the NPs with the highest binding affinity were evaluated for their molecular interactions, including hydrogen bonding and non-covalent interactions, as well as their docking poses. The docking results were visualized using PyMOL and LigPlot+ software to identify and select the best lead compound for further analysis.

### 3.5. Physicochemical and Pharmacokinetic Properties of the Selected Lead Compound

To evaluate the physicochemical properties and toxicity risk parameters of the selected lead compound, its SDF format was retrieved from the PubChem database (PubChem ID: 102365382)(51) and analyzed using the Osiris DataWarrior program. This cheminformatics tool is designed for the in silico evaluation of the physicochemical properties of compounds. The properties examined include molecular weight (MW), LogP, LogS, the number of hydrogen bond donors (HBD), the number of hydrogen bond acceptors (HBA), polar surface area (PSA), and the number of rotatable bonds (Rb). Additionally, the Osiris DataWarrior program can predict toxicity risk parameters such as mutagenic and tumorigenic effects, reproductive effects, and irritating effects.

Furthermore, the pharmacokinetic properties, including Absorption, Distribution, Metabolism, and Excretion (ADME), were analyzed using pkCSM software (http://biosig.unimelb.edu.au/pkcsm). Key pharmaceutical properties, such as Caco2 permeability, skin permeability, human intestinal absorption, blood-brain barrier (BBB) permeability, and central nervous system (CNS) permeability, were assessed. The software also predicts the metabolism of compounds by evaluating descriptors like cytochrome P (CYP) substrates and inhibitors. Additionally, it forecasts the drug's renal excretion using descriptors such as renal organic cation transporter 2 (OCT2), a renal uptake transporter.

### 3.6. Molecular Dynamics Simulations

Molecular dynamic (MD) simulations for protein-compound complexes were performed using Gromacs 2022 (52). Topology files for the protein targets were prepared using pdb2gmx and the CHARM force field. Additionally, topology files for the selected compounds were generated using the CGenFF server (53). The MDs method followed the procedures outlined in previous studies (24, 54). In summary, the equilibration steps were carried out using NVT (constant particle number, volume, and temperature) and NPT (constant particle number, pressure, and temperature) at 300K for 100 ps. Finally, the equilibrated system was subjected to MDs for a duration of 100 ns. The results from the MDs were assessed using various parameters, including root mean square deviation (RMSD), radius of gyration (Rg), protein root mean square fluctuation (RMSF), and hydrogen bonds (H-bonds).

## 4. Results

### 4.1. Analysis of the Structure of Wild-Type and Variants of SARS-CoV-2 RBD

It is well established that a critical step in SARS-CoV-2 infection is the binding of the RBD of the SARS-CoV-2 S protein to the human ACE2 protein. Therefore, mutations in this domain can significantly enhance the binding affinity of RBD to ACE2, while simultaneously reducing the effectiveness of vaccine-induced antibodies. These mutations may also contribute to increased transmission rates and pathogenicity of the SARS-CoV-2 virus. The most common variants of SARS-CoV-2 that have strengthened infectivity are Alpha, Beta, Delta, Gamma, Lambda, Omicron/BA.1, and Omicron/BA.2.

Thus, the primary, secondary, and tertiary structures of the wild-type and variants of SARS-CoV-2 RBD were evaluated and compared. The physicochemical properties of the primary amino acid sequence for both the wild-type and variants were analyzed using Expasy’s ProtParam (http://web.expasy.org/protparam/). The calculated pI values for the wild-type and variants showed that the wild-type RBD has an approximately neutral character, while all variants exhibit a basic character, except for the Lambda variant, which has an acidic character. The instability index (II < 23) for the wild-type and variants indicated that these proteins are likely stable under physiological conditions. More information about the physicochemical properties of the wild-type and variants is provided in Table 2. 

**Table 2.** Comparison of Physiochemical Properties of Wild-Type and Variants of SARS-CoV-2 Receptor-Binding Domain ^a^

Protein	Length	MW, D	pI	-R	+R	GRAVY	Aliphatic Index	Instability Index
**Wild-type**	193	21700.47	7.63	16	17	-0.207	67.62	20.30
**Alpha**	193	21758.64	8.37	15	18	-0.202	62.62	19.55
**Beta**	193	21763.57	8.37	15	18	-0.237	66.11	22.67
**Delta**	193	21769.54	8.38	15	18	-0.266	66.11	20.48
**Lambda**	193	21640.33	6.43	16	16	-0.223	67.62	21.19
**Omicron/BA.1**	193	21877.81	8.85	15	21	-0.228	68.13	18.26
**Omicron/BA.2**	193	21789.75	8.84	15	21	-0.221	68.13	18.21

Abbreviations: D, daltons; -R, negative-charged residues (Asp and Glu); +R, positive-charged residues (Arg and Lys); GRAVY, grand average of hydropathicity.

^a^ A pI > 7 indicates basic character, while a pI < 7 indicates acidic character. An instability index (II < 23) indicates that the protein is stable under physiological conditions.

Analysis of the secondary structures of the wild-type and variants of SARS-CoV-2 RBD revealed that all forms are primarily composed of coiled-coil structures. However, the largest number of residues engaged in the formation of helix and β-strand conformations were found in Lambda, Omicron/BA.2, and wild-type, with Lambda having the highest number, followed by Omicron/BA.2 (Table 3). 

**Table 3.** Comparison of the Secondary Structure Characteristics of Wild-Type and Variants of SARS-CoV-2 Receptor-Binding Domain ^a^

Protein	Helix	Beta	Coil
**Wild-type**	7.98	39.36	52.66
**Alpha**	7.98	38.83	53.19
**Beta**	7.98	37.23	54.79
**Delta**	7.98	37.23	54.79
**Lambda**	9.04	39.36	51.60
**Omicron/BA.1**	7.45	38.30	54.26
**Omicron/BA.2**	9.04	38.83	52.13

^a^ Values are expressed as %.

**Figure 1.**
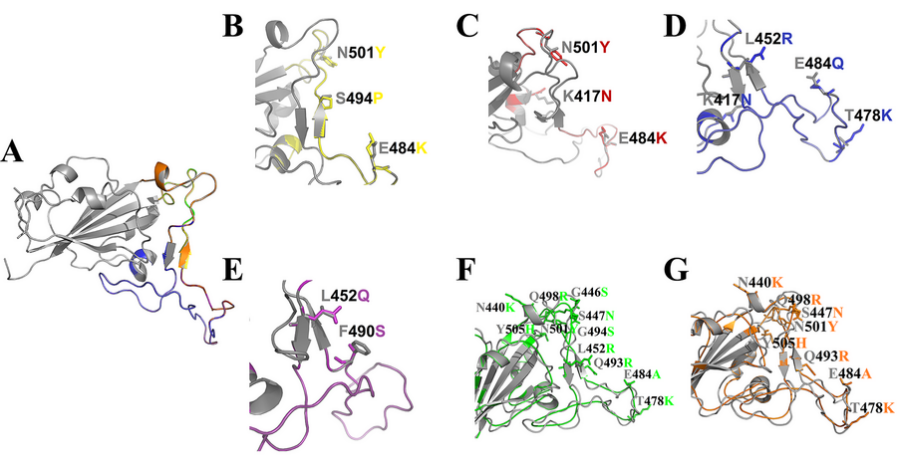
Structural alignment of wild-type and variants of SARS-CoV-2 RBD. Cartoon representation of the wild-type protein (A), Alpha (B), Beta (C), Delta (D), Lambda (E), Omicron/BA.1 (F), Omicron/BA.2 (G) variants are shown in gray, yellow, red, blue, purple, green, and orange, respectively, along with the mutated residue names. These residues are represented as A (Ala), R (Arg), N (Asn), C (Cys), Q (Gln), E (Glu), G (Gly), H (His), L (Leu), K (Lys), F (Phe), P (Pro), S (Ser), T (Thr), Y (Tyr).

Models of the variants were generated using homology, and the best 3D structure was subsequently refined using the GalaxyRefine web tool. Quality evaluation of the final models confirmed their suitability for further computational research (Appendix 1 in Supplementary File). The Ramachandran plots and the stereochemical quality of all modeled 3D structures were evaluated using PROCHECK, which showed that a satisfactory proportion of residues were located in the most favored regions. Moreover, the comparison of the modeled 3D structures with the wild-type revealed no significant structural differences between the variants and the wild-type structures (Figure 1). Additionally, the total charge of the modeled 3D structures and the wild-type was calculated as follows: Wild-type: 0.00, Alpha: 2.00, Beta: 2.00, Delta: 2.00, Lambda: -1.00, Omicron/BA.1: 4.00, Omicron/BA.2: 4.00.

### 4.2. Evaluation of Structure-Based Virtual Screening Results

Structure-based virtual screening for potential NPs from the StreptomeDB database was performed against the wild-type and Alpha, Beta, Delta, Lambda, Omicron/BA.1, and Omicron/BA.2 variants of SARS-CoV-2 S-RBD. A total of 6,524 NPs from *Streptomyces* spp. were docked to the prepared protein targets using Smina software. The top 20 NPs with the highest binding affinity were identified at the binding sites of the wild-type and variants. These compounds were clustered based on structural similarity using ChemMine Web Tools (http://chemmine.ucr.edu/) (55) to exclude compounds with identical structures. Ultimately, 5 NPs with the highest binding affinity and best conformations were selected as hit compounds for both wild-type and variants (Table 4). The 2D structure of some of the hit compounds is shown in Appendix 2 in Supplementary File.

**Table 4.** Protein-Compound Docking Results Using Smina Software

Wild-type Binding Affinity, Kcal/mol		Alpha Mutant Binding Affinity, Kcal/mol		Beta Mutant Binding Affinity, Kcal/mol		Delta Mutant Binding Affinity, Kcal/mol		Lambda Mutant Binding Affinity, Kcal/mol		Omicron/BA.1 Mutant Binding Affinity, Kcal/mol		Omicron/BA.2 Mutant Binding Affinity, Kcal/mol	
**Stambomycin_B **	-11.60	Plicamycin	-11.82	Stambomycin_B	-12.00	Stambomycin_B	-12.47	Stambomycin_B	-11.64	Stambomycin_B	-12.56	Plicamycin	-11.46
**ActinomycinY8**	-10.27	Ristocetin A	-11.51	Actinomycin Y6	-10.50	Langkocycline_B2	-11.07	Ristocetin_A	-10.54	N,C7-Dixiamycin	-11.16	Stambomycin_B	-11.44
**Actinomycinzp**	-9.85	Stambomycin_B	-10.60	LangkocyclineB1	-10.39	Val-geninthiocin	-10.42	Actinomycin Y8	-9.65	Langkocycline_B1	-11.15	Ristocetin_A	-10.35
**Val-geninthiocin**	-9.71	Gilvusmycin	-9.92	Pyrroindomycin A	-9.68	Actinomycin_Y6	-10.40	Rapamycin	-9.49	Rapamycin	-11.14	N,C7-Dixiamycin	-9.85
**N,C7-Dixiamycin**	-9.43	Rapamycin	-9.69	Lobophorin	-9.58	Pyrroindomycin_A	-10.25	Val-geninthiocin	-9.38	Actinomycin ZP	-10.90	Actinomycin ZP	-9.71

**Figure 2.**
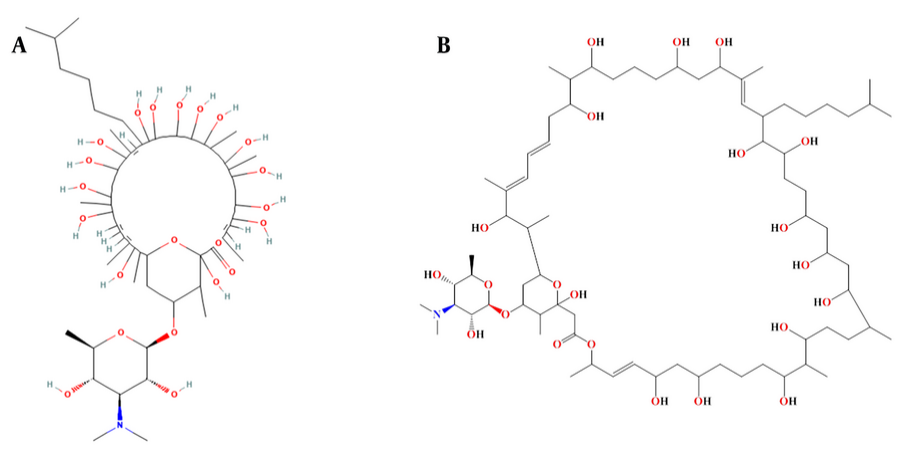
The 2D structure of the selected lead compound. A, the 2D representation of 'Stambomycin B' was prepared from the PubChem Database; B, another 2D representation of 'Stambomycin B' was made more clearly using ChemDraw software.

These top 5 compounds were further investigated to identify those capable of inhibiting both the wild-type and variants in the panel. As shown in the results, 'Stambomycin B' exhibited the highest binding affinity compared to the other compounds. The complex of 'Stambomycin B' with the wild-type, Alpha, Beta, Delta, Lambda, Omicron/BA.1, and Omicron/BA.2 variants showed binding affinities of -11.60 Kcal/mol, -10.60 Kcal/mol, -12.00 Kcal/mol, -12.47 Kcal/mol, -11.64 Kcal/mol, -12.56 Kcal/mol, and -11.44 Kcal/mol, respectively. Not only did 'Stambomycin B' exhibit the highest binding affinity, but it was also able to inhibit the wild-type and variants. Therefore, 'Stambomycin B' was selected as the best lead compound for further analysis. Figure 2 shows the 2D structure of 'Stambomycin B'.

Additionally, we conducted an analysis of the interactions between the ‘Stambomycin B’ complex and both the wild-type and variants using PyMOL and LigPlot software.

**Figure 3.**
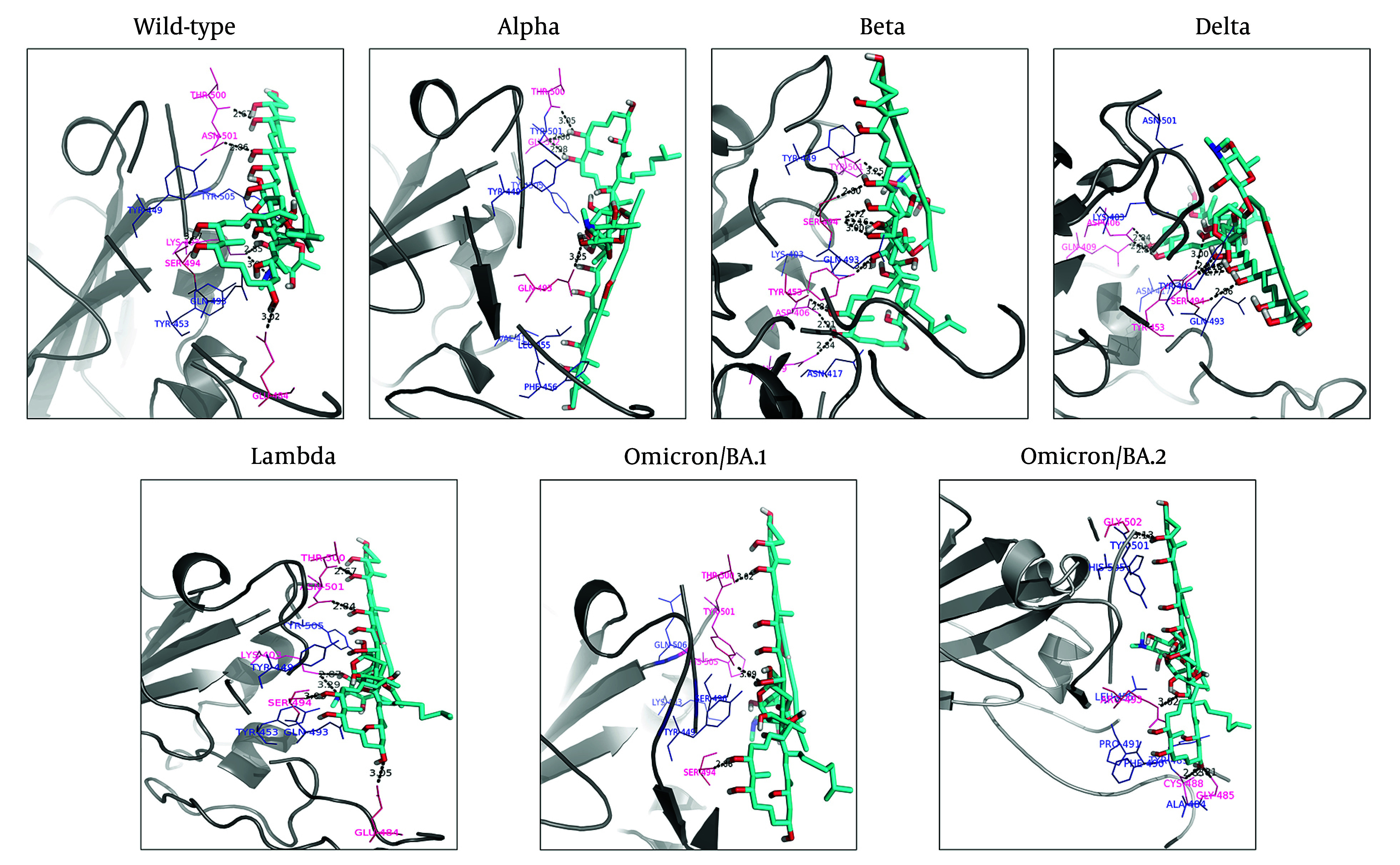
Binding orientations of residues within the binding sites of the wild-type and variants of SARS-CoV-2 RBD during interactions with ‘Stambomycin B’. The 3D structures of the wild-type and variants of SARS-CoV-2 RBD are shown in gray (cartoon representation). The 3D structure of ‘Stambomycin B’ is shown in cyan (stick representation). The residues involved in hydrogen bonds and hydrophobic interactions are shown in pink and blue (line representation), respectively. Hydrogen bonds in the protein-ligand complex are depicted as black dotted lines.

The results showed that six hydrogen bonds were formed between the hydroxyl and nitro groups of ‘Stambomycin B’ and residues Lys403, Glu484, Ser494, Thr500, and Asn501 of the wild-type. Hydrophobic interactions were observed between the ligand and residues Tyr449, Gln493, Tyr453, and Tyr505 (Figure 3 - Wild-type).

Upon assessing the Alpha-Stambomycin B complex, it was found to form four hydrogen bonds between the hydroxyl groups of ‘Stambomycin B’ and residues Gln493, Thr500, and Gly502. Hydrophobic interactions were also observed between ‘Stambomycin B’ and residues Val417, Leu455, Tyr449, Phe456, Thr500, Tyr501, Gly502, and Tyr505 (Figure 3 - Alpha).

The analysis of the Beta-Stambomycin B complex showed that nine hydrogen bonds were formed between the hydroxyl group of ‘Stambomycin B’ and the residues Asp406, Gln409, Tyr453, Ser494, and Tyr501. Additionally, the residues Lys403, Asn417, Tyr449, and Gln493 formed hydrophobic interactions with ‘Stambomycin B’ (Figure 3 - Beta).

Based on the docking results, eight hydrogen bonds were formed between ‘Stambomycin B’ and the Delta variant, including the residues Asp406, Gln409, Tyr453, and Ser494 in the binding site, with the hydroxyl groups of ‘Stambomycin B’. Furthermore, the residues Lys403, Asn417, Tyr449, Gln493, and Asn501 formed hydrophobic interactions with ‘Stambomycin B’ (Figure 3 - Delta).

In addition, six hydrogen bonds were formed between the hydroxyl and nitro groups of ‘Stambomycin B’ and the residues Lys403, Glu484, Ser494, Thr500, and Asn501 of the Lambda variant. The residues Tyr449, Tyr453, Gln493, and Tyr505 also formed hydrophobic interactions with ‘Stambomycin B’ (Figure 3 - Lambda).

According to the docking results, three hydrogen bonds were formed between ‘Stambomycin B’ and the Omicron/BA.1 variant, including the residues Ser494, Thr500, and Tyr501, with the hydroxyl groups of ‘Stambomycin B’. Additionally, the residues Lys403, Tyr449, Arg493, Ser496, and His505 formed hydrophobic interactions with ‘Stambomycin B’ (Figure 3 Omicron/BA.1).

The Omicron/BA.2-Stambomycin B complex formed four hydrogen bonds between the hydroxyl and nitro groups of ‘Stambomycin B’ and the residues Gly502, Arg493, Cys488, and Gly485. Moreover, the residues Leu455, Ala484, Tyr489, Phe490, Tyr501, and His505 formed hydrophobic interactions with ‘Stambomycin B’ (Figure 3 Omicron/BA.2). More details on the interactions between ‘Stambomycin B’ and the wild-type and variants are provided in Appendix 3 in Supplementary File.

### 4.3. Assessment of the Physicochemical and Pharmacokinetic Properties of the Selected Lead Compound

It is well documented that Lipinski's rule of five (RO5) is essential for identifying potential hits and leads, with 90% of orally active compounds typically meeting these criteria. Rule of five stipulates that an orally active compound should have a molecular weight under 500 Da, an XlogP (octanol-water partition coefficient) below 5, fewer than 10 hydrogen bond acceptors, and fewer than 5 hydrogen bond donors (56). Silvio Roggo has noted that some NPs, despite not adhering to RO5, have shown promise as effective drugs. Consequently, he proposed that NPs can be exempted from RO5 (57).

However, the prediction of the physicochemical properties and toxicity risk parameters of the selected lead compound, ‘Stambomycin B’, using the Osiris DataWarrior program revealed the following properties: Molecular weight = 1378.8 g/mol, XlogP = 7.833, number of hydrogen bond acceptors = 23, number of hydrogen bond donors = 17, polar surface area = 401 Å², and rotatable bonds = 8. Additionally, the toxicity profiles of ‘Stambomycin B’ were evaluated, and the results showed the following: Mutagenic = none, tumorigenic = none, reproductive effect = none, and irritating effect = none. Consequently, these properties indicate that ‘Stambomycin B’ is a safe compound with a favorable drug profile.

Additionally, the pharmacokinetic properties of 'Stambomycin B' were predicted using the pkCSM tool. To assess compound absorption, we predicted Caco-2 permeability (LogPapp), skin permeability (Logkp), and human intestinal absorption (HIA). According to the pkCSM software, a compound is expected to have high Caco-2 permeability and relatively low skin permeability if LogPapp is greater than 0.90 and Logkp is greater than -2.5. Moreover, if intestinal absorption is less than 30%, the compound is considered poorly absorbed. The results indicated LogPapp at 0.156, Logkp at -2.735, and HIA at 0.000. Therefore, 'Stambomycin B' exhibits low Caco-2 permeability, relatively low skin permeability, and no human intestinal absorption.

To study compound distribution, we predicted blood-brain barrier (BBB) permeability (LogBB) and CNS permeability (LogPS). According to the software, a compound with LogBB greater than 0.3 can cross the BBB, while a compound with LogBB less than -1 has poor distribution to the brain. Additionally, a compound with LogPS greater than -2 and LogPS less than -3 is considered penetrable and impenetrable to the CNS, respectively. Our results showed LogBB at -2.4188 and LogPS at -4.371, indicating that 'Stambomycin B' cannot cross the BBB and lacks the capability to penetrate the CNS.

To investigate compound metabolism, we predicted the involvement of CYP enzymes and found that 'Stambomycin B' is neither a substrate nor an inhibitor of CYP enzymes. Furthermore, we analyzed compound excretion via OCT2, which showed that 'Stambomycin B' is not excreted through the kidneys.

### 4.3. Evaluation of Molecular Dynamics Simulations

**Figure 4.**
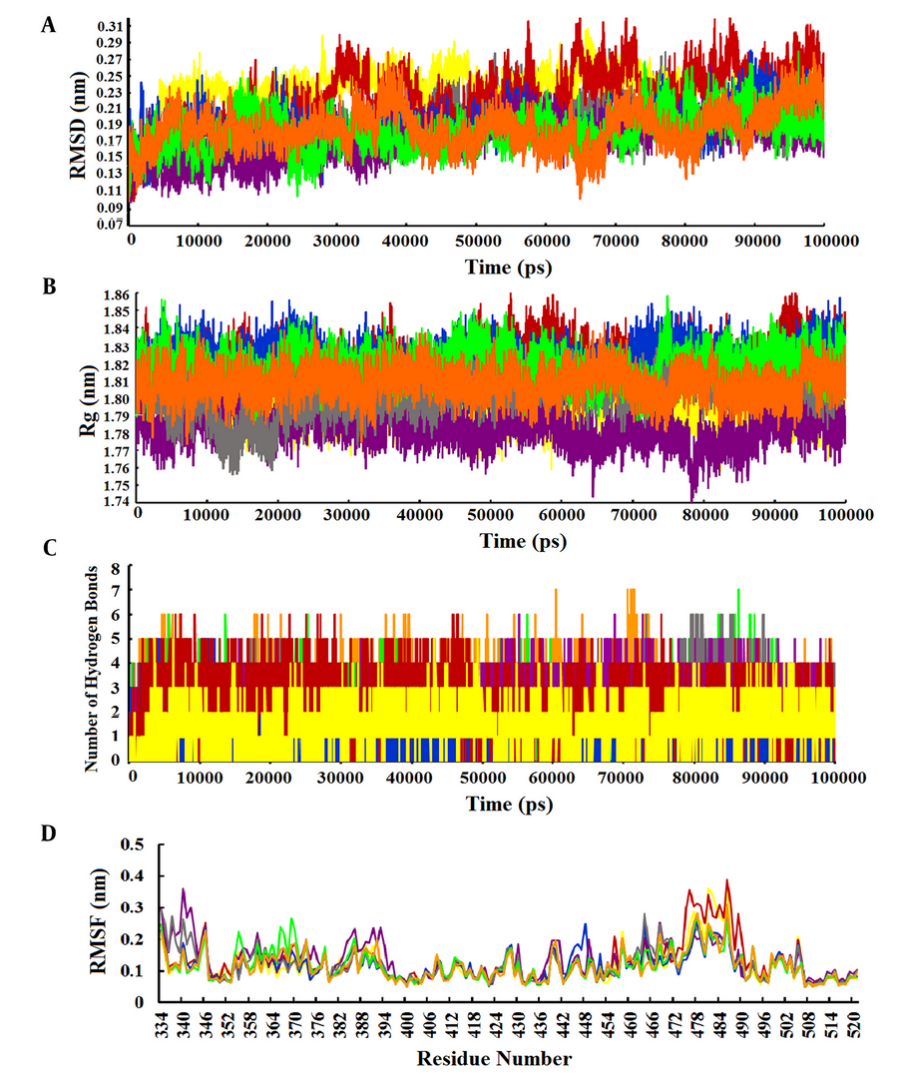
Analysis of MD simulation results. A, RMSD plots of the complexes of wild-type and variants of SARS-CoV-2 RBD with ‘Stambomycin B’ during 100 ns of simulations; B, Rg plots of the complexes of wild-type and variants of SARS-CoV-2 RBD with ‘Stambomycin B’ during 100 ns of simulations; C, the number of H-bonds between wild-type and variants of SARS-CoV-2 RBD with ‘Stambomycin B’ during 100 ns of simulations; D, RMSF of backbone Cα atoms of the wild-type and variants of SARS-CoV-2 RBD versus residue number in the sequence. In all plots, the colors represent wild-type (gray), Alpha (yellow), Beta (red), Delta (blue), Lambda (purple), Omicron/BA.1 (green), and Omicron/BA.2 (orange).

The RMSD value was calculated to evaluate the conformational changes and stability of the complex system. The RMSD value for the wild-type-Stambomycin B complex ranged from 0.10 to 0.28 nm, while the RMSD values for the complexes with Alpha, Beta, Delta, and Lambda fluctuated between 0.10 - 0.30 nm, 0.10 - 0.31 nm, 0.10 - 0.27 nm, and 0.10 - 0.26 nm, respectively. Additionally, the RMSD values for the complexes of Omicron/BA.1 and Omicron/BA.2 with Stambomycin B displayed nearly identical patterns of RMSD changes, ranging from approximately 0.10 to 0.27 nm during the 100 ns simulations. A more detailed analysis of the RMSD values showed that the wild-type, Delta, Lambda, and Omicron/BA.1 complexes exhibited higher stability during the 100 ns simulation, while the Alpha, Beta, and Omicron/BA.2 variants displayed greater fluctuations during the same period (Figure 4A). 

In parallel, the Rg value was calculated as a parameter of stability and protein structure compactness. As shown in Figure 4B, the Rg values for the complexes of wild-type and Alpha with ‘Stambomycin B’ ranged from 1.76 to 1.84 nm. The complexes of Beta and Delta ranged from 1.76 to 1.86 nm, while Lambda ranged from 1.73 to 1.81 nm. The complexes of Omicron/BA.1 and Omicron/BA.2 ranged from 1.79 to 1.85 nm and 1.74 to 1.87 nm, respectively, during the 100 ns simulation period. Based on the results, the complexes of ‘Stambomycin B’ with wild-type, Delta, Lambda, Omicron/BA.1, and Omicron/BA.2 exhibited higher compactness during the 100 ns simulations. In contrast, the complexes of Alpha and Beta with ‘Stambomycin B’ showed slight fluctuations throughout the simulation period.

Although the previous analyses indicated a stable and flexible conformation of the complexes, we also conducted an H-bond analysis to support our earlier findings. The results of the H-bond analysis between ‘Stambomycin B’ and the variants during MD simulations showed that the complexes of wild-type and variants with ‘Stambomycin B’ possessed approximately 0-6 H-bonds. The Beta, Lambda, Omicron/BA.1, and Omicron/BA.2 complexes had around 1-5 H-bonds, while Alpha and Delta showed around 0-3 H-bonds (Figure 4C). 

To study the flexibility and dynamics of the protein structure in complexes, the RMSF was calculated. The RMSF values for the complexes revealed that all residues fluctuated between 0.05 and 0.38 nm, with the values for the complexes of wild-type and Omicron/BA.1 ranging from 0.05 to 0.27 nm during the 100 ns simulation. Additionally, the RMSF value for the complexes of Alpha and Lambda ranged from 0.05 to 0.36 nm. The Beta-Stambomycin B, Delta-Stambomycin B, and Omicron/BA.2-Stambomycin B complexes fluctuated from 0.05 to 0.38 nm, 0.05 to 0.24 nm, and 0.05 to 0.31 nm, respectively. The RMSF values of the complexes showed that all residues in the binding pocket of wild-type and variants (residues 449 to 505) fluctuated between 0.05 nm and 0.38 nm, indicating that these residues can stably interact with ‘Stambomycin B’ throughout the MD simulations (Figure 4D). 

## 5. Discussion

As a result of the spread of SARS-CoV-2, new variants have emerged, and it is believed that some of the SARS-CoV-2 S protein variants have a strong affinity for the ACE2 receptor. The emerging SARS-CoV-2 variants could hinder researchers’ efforts, as all vaccine targets are based on the wild-type and some variants. Drug discovery is a lengthy, costly, and complex process. However, the time required to find the optimal agent for the chosen target can be shortened by virtual screening of relevant databases and the application of modern bioinformatics and cheminformatics approaches. The virtual screening process has become the gold standard for the preliminary phase of drug development.

This study provides computational insights into the structural alterations in the SARS-CoV-2 RBD induced by the variants. The analysis showed that the SARS-CoV-2 RBD in the Omicron variant has more variable residues compared to the wild-type variant (Table 1). The study of the physicochemical properties of the primary structures of the SARS-CoV-2 RBD from both the wild-type and variants, as well as their secondary structures, revealed that the Lambda variant differs from the other variants (Tables 2 and 3). Subsequently, the 3D structures of the variants were predicted. The assessment of the stereochemical quality of the models indicated their suitability (Appendix 1 in Supplementary File). Superimposition of all variants with the wild-type revealed no significant structural differences (Figure 1). 

To identify potential NPs and inhibit the interaction between SARS-CoV-2 RBD and ACE2, the SBVS method and molecular docking studies were performed between the flexible residues of the selected binding site of SARS-CoV-2 RBD (both wild-type and variants) and NPs from the StreptomeDB library.

A more detailed analysis of the hit compounds revealed that ‘Stambomycin B’ exhibited the highest binding affinity with the wild-type and variants compared to the other hit compounds, with binding affinities ranging from -10.60 to -12.47 kcal/mol (Table 4). 

‘Stambomycin B’ is a macrolide compound produced by *Streptomyces ambofaciens*. It is well documented that *S. ambofaciens* produces two antibiotics: The macrolide spiramycin, which is used to treat bacterial infections and toxoplasmosis, and the pyrrolamide congocidine (58). Genome sequencing analysis of *S. ambofaciens* has shown that it contains several gene clusters responsible for the biosynthesis of secondary metabolites (59). Among these, one of the most significant and largest gene clusters is the cryptic type I modular polyketide synthase (PKS), consisting of 25 genes (nine of which are involved in encoding PKSs) (60). Polyketides include various chemical classes such as macrolides, polyenes, aromatics, and polyethers. Interestingly, these compounds are used as antibiotics, antitumor agents, immunosuppressants, and cholesterol-lowering drugs (61). Polyketide synthase is responsible for the production of stambomycins A, B, C, and D. ‘Stambomycin B’ is a metabolic product of the PKS gene cluster, containing 231 carbon bonds, multiple double bonds, hydroxyl groups, and ether bonds, with the chemical formula C73H133NO22. The main functional groups of ‘Stambomycin B’ include 17 hydroxyl groups, 16 secondary alcohols, 3 ether groups, 1 ester group, and 1 tertiary amine. The genes responsible for the biosynthesis of stambomycin B are clustered in the genome of *S. ambofaciens*, and its biosynthesis begins with the assembly of the polyketide chain by the PKS complex. A unique feature of the biosynthesis of ‘Stambomycin B’ is the formation of its large lactone ring. Once the core structure is formed, various tailoring enzymes, including glycosyltransferases and hydroxylases, modify the molecule to generate the final active compound (62).

The docking poses and interacting residues of the wild-type and variants with ‘Stambomycin B’ are shown in Figure 3 and Appendix 3 in Supplementary File. As mentioned previously, ‘Stambomycin B’ is a large compound with many rotatable bonds, enabling it to form numerous hydrogen and hydrophobic bonds with residues at the binding sites of both the wild-type and variants. Most of these interactions arise from the compound's many hydroxyl groups, which are located in the macrolide ring.

The docking results indicate that this inhibitor can interact not only with residues in the binding sites (Tyr449, Tyr453, Leu455, Phe456, Ala475, Gly476, Phe486, Asn487, Tyr489, Gln493, Gly496, Gln498, Thr500, Asn501, Gly502, and Tyr505) but also with several additional residues, including Lys403, Asp406, Gln409, Asn417, Ala484, Gly485, Cys488, Phe490, Ser494, and Gly502. Notably, the residues Lys403, Tyr449, Ser494, Gln493, Thr500, Asn501, and Tyr505 were the most frequently involved in interactions across the complexes.

One important parameter for evaluating a protein-ligand complex is the root mean square deviation (RMSD) of the Cα atoms in the protein backbone. This metric reflects the conformational stability of the protein during dynamic simulations. A system is considered equilibrated and stable when it exhibits low RMSD levels with consistent fluctuations throughout the simulation. In contrast, higher fluctuations indicate lower stability (19). In our analysis, we found that the minimum and maximum RMSDs for the complexes ranged from 0.100 to 0.315 nm. The RMSD values demonstrated stable trajectories with minor fluctuations, suggesting that the protein backbone is generally stable. We also observed some fluctuations at different time points (Figure 4A). However, the RMSD results indicated that the complexes of ‘Stambomycin B’ with Delta, Lambda, and Omicron/BA.1 exhibited the lowest RMSD values and fluctuations compared to the other complexes, confirming their higher stability and fewer conformational changes.

The radius of gyration (Rg) is a parameter used to calculate the compactness and folding of a protein structure. It is defined as the mean square distance of each atom in the protein from the center of mass. This value provides a quantitative assessment of the overall size and shape of the protein. In general, proteins with lower Rg values are more compact, while proteins with higher Rg values are more flexible (63). Our results showed that the Rg values ranged from 1.73 to 1.87 nm. All complexes exhibited similar patterns of Rg value changes and remained very compact during the 100 ns simulations, except for the complexes of Alpha and Beta with 'Stambomycin B', which showed slight fluctuations (Figure 4B). 

Another important parameter for evaluating a protein-ligand complex is the analysis of hydrogen bonds between the ligand and the protein, which helps maintain a compact and well-oriented structure. Additionally, the flexibility of the protein residues is crucial for forming bonds with the ligand molecules (19). The results of the hydrogen bond analysis indicated that both the wild-type and variants formed strong and stable bindings with 'Stambomycin B' during the simulation period (Figure 4C). 

The RMSF is a parameter used to evaluate protein residues that are crucial for achieving a stable conformation in a protein-ligand complex. The RMSF analyzes specific parts of the protein that deviate from their average structure, typically due to ligand interaction. The fluctuations observed for each residue indicate its degree of flexibility. Therefore, residues with higher RMSF values show greater flexibility, which correlates with an increased potential to interact with the ligand molecule. Conversely, lower RMSF fluctuations indicate lower flexibility and, consequently, a reduced interaction potential (19). The results of the RMSF analysis of the complexes showed that the overall RMSF was low (< 0.4 nm), indicating stable interactions within the complexes (Figure 4D). However, notable peaks with increased fluctuations were observed at certain residues, particularly in the binding pocket (residues 449 to 505), indicating enhanced interaction potential. This suggests that the ligands in the protein's binding pocket can adapt effectively.

In conclusion, it appears that 'Stambomycin B' has the potential to be a candidate NP for overcoming all mutants that may arise in the binding of SARS-CoV-2 RBD to ACE2, including those that may emerge in the future. Additionally, it can be used for further studies aimed at identifying new drugs against SARS-CoV-2.

### 5.1. Conclusions

In this study, a potential lead natural product was identified through SBVS from the StreptomeDB library. Molecular docking was performed between the StreptomeDB library and the structures of the wild-type SARS-CoV-2 RBD (PDB ID: 6VW1), as well as the Alpha, Beta, Delta, Lambda, Omicron/BA.1, and Omicron/BA.2 variants of the SARS-CoV-2 RBD. The molecular docking results indicated that ‘Stambomycin B’ exhibited better binding affinity than other NPs for both the wild-type and the variants. Subsequently, MD simulations were conducted for the complexes of the proteins (wild-type and variants) with ‘Stambomycin B’ over 100 ns. The results showed that ‘Stambomycin B’ formed stable complexes with both the wild-type and variants of SARS-CoV-2 RBD during the simulation period. Based on these in silico investigations, it can be concluded that ‘Stambomycin B’ can inhibit the interaction of SARS-CoV-2 RBD with ACE2. Furthermore, ‘Stambomycin B’ has the potential to effectively combat all mutants that may arise in the binding of SARS-CoV-2 RBD to ACE2, including those that may emerge in the future. However, the obtained results should be further investigated through in vitro and in vivo assessments, and they may also provide valuable insights for future studies.

ijpr-23-1-150879-s001.pdf

## Data Availability

The datasets generated and/or analyzed during the current study are available from the corresponding author upon reasonable request.
