# Supplementary material for: Predictive Insights Into Bioactive Compounds from Streptomyces as Inhibitors of SARS-CoV-2 Mutant Strains by Receptor Binding Domain: Molecular Docking and Dynamics Simulation Approaches
Source: Iran J Pharm Res. 2024 Dec 8;23(1):e150879. doi: 10.5812/ijpr-150879 (PMC11892749; doi:10.5812/ijpr-150879)
Supplement: ijpr-23-1-150879-s001.pdf [file ijpr-23-1-150879-s001.pdf]

Appendix 1. Comparison of validation of predicted 3D models of variants of SARS-CoV-2 RBD with wild-type by different scores

Protein	Ramachandran plot quality (%)				Verify3D (%)	ERRAT (%)
	Most favored	Additionally allowed	Generously allowed	Disallowed		
Wild-type	83.7	15.7	0.0	0.6	91.71	90.909
Alpha	83.6	15.8	0.0	0.6	91.71	87.500
Beta	83.7	15.7	0.0	0.6	92.23	87.500
Delta	83.7	15.7	0.0	0.6	92.23	90.909
Lambda	83.7	15.7	0.0	0.6	92.75	90.909
Omicron/BA.1	82.7	16.1	0.0	1.2	92.23	85.227
Omicron/BA.2	83.7	15.7	0.0	0.6	92.75	85.795

Appendix 2

Actinomycin Y8

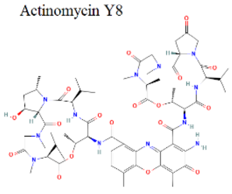

Actinomycin Zp

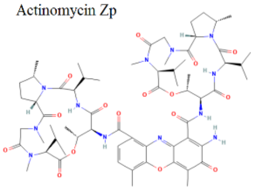

Val-Geninthiocin

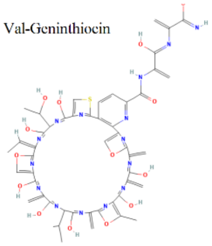

Dixiamycin

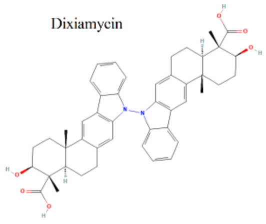

Plicamycin

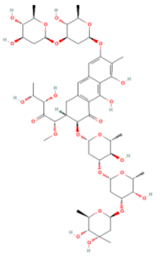

Ristocetin A

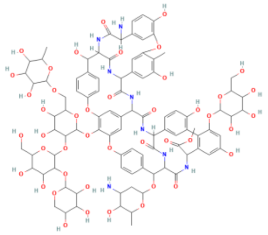

Rapamycin

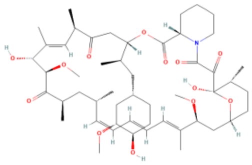

Gilvusmycin

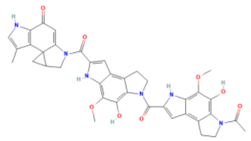

Actinomycin Y6

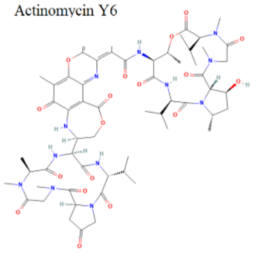

Langkocycline B1

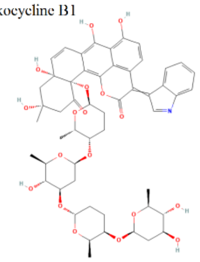

Pyrroindomycin A

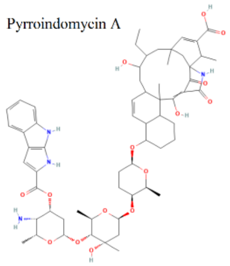

Lobophorin

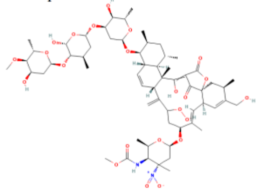

Appendix 3. Evaluation binding affinity and interaction summary of 'Stambomycin B' with wild-type and variants.

Protein	Stambomycin_B			
	**Binding affinity (Kcal/ mol)**	**HB-AAs[a]**	**angle / Bond length**	**NH-AAs[b]**
**Wild-type**	**-11.60**	**Lys403**<br>**Lys403**<br>**Glu484**<br>**Ser494**<br>**Thr500**<br>**Asn501**	**N—H—O = 48.25° / 2.85 Å**<br>**N—H—N = 59.18°/ 3.25 Å**<br>**O—H—O = 83.81°/ 3.02 Å**<br>**O—H—O = 159.84°/ 3.07 Å**<br>**O—H—O = 59.46°/ 2.67 Å**	**Tyr449**<br>**Gln493**<br>**Tyr453**<br>**Tyr505**

			N —H—O = 78.86°/ 2.86 Å	
Alpha	-10.60	Gln493<br><br>Thr500<br><br>Gly502	N—H—O = 154.58°/ 3.25 Å<br><br>O—H—O = 115.28°/ 3.05 Å<br><br>N—H—O = 19.78°/ 2.86 Å	Val417<br><br>Leu455<br><br>Tyr449<br><br>Phe456<br><br>Thr500<br><br>Tyr501<br><br>Gly502<br><br>Tyr505

**Beta**	**-12.00**	**Asp406**<br>**Asp406**<br>**Gln409**<br>**Tyr453**<br>**Ser494**<br>**Ser494**<br>**Ser494**<br>**Tyr501**	**O—H—O =135.40°/ 2.84 Å**<br>**O—H—O =150.12°/ 2.91 Å**<br>**N —H—O =78.34°/ 2.84 Å**<br>**O—H—O = 91.06°/ 3.01 Å**<br>**O—H—O = 75.77°/ 2.72 Å**<br>**O—H—O = 40.33°/ 3.16 Å**<br>**O—H—O = 80.23°/ 3.00 Å**<br>**O—H—O = 68.45°/ 3.25 Å**	**Lys403**<br>**Asn417**<br>**Tyr449**<br>**Gln493**
		**Asp406**	**O—H—O = 147.09°/ 2.86 Å**	**Lys403**

**Delta**	**-12.47**	**Asp406**<br><br>**Gln409**<br><br>**Tyr453**<br><br>**Ser494**<br><br>**Ser494**<br><br>**Ser494**	**O—H—O = 103.01°/ 2.84 Å**<br><br>**N—H—O = 124.06°/ 2.81 Å**<br><br>**O—H—O = 65.30°/ 3.00 Å**<br><br>**O—H—O =89.70°/ 2.86 Å**<br><br>**O—H—O =85.05°/ 2.94 Å**<br><br>**O—H—O =85.05.33°/ 2.94 Å**	**Asn417**<br><br>**Tyr449**<br><br>**Gln493**<br><br>**Asn501**
		**Lys403**<br><br>**Lys403**<br><br>**Glu484**	**N—H—O =117.19°/ 2.87 Å**<br><br>**N—H—N =134.84°/ 3.29 Å**	**Tyr449**<br><br>**Tyr453**<br><br>**Gln493**

**Lambda**	**-11.64**	**Ser494**<br><br>**Thr500**<br><br>**Asn501**	**O—H—O =76.66°/ 3.05 Å**<br><br>**O—H—O =97.02°/ 3.09 Å**<br><br>**O—H—O =102.34°/ 2.67 Å**<br><br>**N—H—O =85.93°/ 2.84 Å**	**Tyr505**
**Omicron/BA.1**	**-12.56**	**Ser494**<br><br>**Thr500**<br><br>**Tyr501**	**O—H—O =169.63°/ 2.88 Å**<br><br>**O—H—O =130.63°/ 3.02 Å**<br><br>**O—H—O =130.68°/ 3.02 Å**	**Lys403**<br><br>**Tyr449**<br><br>**Arg493**<br><br>**Ser496**<br><br>**His505**

**Omicron/BA.2**	**-11.44**	**Gly485**<br>**Cys488**<br>**Arg493**<br>**Gly502**	**O—H—O =79.46°/ 2.81 Å**<br>**O—H—O =128.69°/ 2.83 Å**<br>**N—H—O =53.9°/ 3.02 Å**<br>**N—H—O =104.10°/ 3.13 Å**	**Leu455**<br>**Ala484**<br>**Tyr489**<br>**Phe490**<br>**Tyr501**<br>**His505**
**[a] Hydrogen bonds forming Amino Acids, [b] Hydrophobic bonds forming Amino Acids,**				
